# Auricular stimulation vs. expressive writing for exam anxiety in medical students – A randomized crossover investigation

**DOI:** 10.1371/journal.pone.0238307

**Published:** 2020-08-27

**Authors:** Taras Usichenko, Anna Wenzel, Catharina Klausenitz, Astrid Petersmann, Thomas Hesse, Nicola Neumann, Klaus Hahnenkamp

**Affiliations:** 1 Department of Anesthesiology, University Medicine of Greifswald, Greifswald, Germany; 2 Department of Anesthesia, McMaster University, Hamilton, Canada; 3 Institute of Clinical Chemistry and Laboratory Medicine, University Medicine of Greifswald, Greifswald, Germany; 4 Institute of Diagnostic Radiology and Neuroradiology, Functional Imaging Unit, University Medicine of Greifswald, Greifswald, Germany; University of Eastern Finland, SWEDEN

## Abstract

**Objective:**

Auricular stimulation (AS) is a promising method in the treatment of situational anxiety. Expressive writing (EW) is an established psychological method, which reduces test anxiety and improves exam results. The aim of this crossover trial was to compare AS with EW, and with the no intervention (NI) condition, for treatment of exam anxiety.

**Methods:**

Healthy medical students underwent 3 comparable anatomy exams with an interval of one month, either performing EW, receiving AS or NI prior to the exam; the order of interventions was randomized. AS was applied using indwelling fixed needles bilaterally at the areas innervated mostly by the auricular branch of the vagal nerve on the day before the exam. Anxiety level, measured using State-Trait-Anxiety Inventory (STAI) before and after the interventions and immediately before exam, was the primary outcome. Quality of night sleep, blood pressure, heart rate and activity of salivary alpha-amylase (sAA) were analyzed across 3 conditions.

**Results:**

All 37 included participants completed the study. Anxiety level (STAI) decreased immediately after AS in comparison with baseline (P = 0.02) and remained lower in comparison with that after EW and NI (P<0.01) on the day of exam. After EW and NI anxiety increased on the day of exam in comparison with baseline (P<0.01). Quality of sleep improved after AS in comparison with both control conditions (P<0.01). The activity of sAA decreased after EW and after AS (P<0.05) but not after NI condition.

**Conclusion:**

Auricular stimulation, but not expressive writing, reduced exam anxiety and improved quality of sleep in medical students. These changes might be due to reduced activity of the sympathetic nervous system.

## Introduction

Exam (or test) anxiety, a kind of situational anxiety, is widely spread among university students [1], even reaching the prevalence of 65% among medical students from Taibah University in Saudi Arabia [2]. Exam anxiety influences both physical and psychological conditions of students [3,4], leading to impaired academic performance during the exams [3,5].

Several interventions are known to reduce the fear before exams. Writing about testing worries and cognitive behavioral therapy are the most common effective methods described previously [6,7], whereas expressive writing, beyond reducing exam anxiety, is claimed to improve academic performance [7].

Auricular acupuncture is a complementary medicine technique, which is physiologically based on the mechanical stimulation of auricular branch of vagal nerve, auriculotemporal nerve (from trigeminal nerve and great auricular nerve from the cervical plexus [8]. Auricular acupuncture was shown to reduce situational anxiety in various clinical situations, such as dental treatment and surgery [9,10]. In a recent randomized crossover trial, both acupuncture-like auricular stimulation (AS) and placebo intervention reduced pre-exam anxiety and duration of sleep in medical students, where AS was superior to placebo procedure in treatment of anxiety [11].

In order to further assess the genuine clinical effectiveness of AS in the treatment of pre-exam anxiety in medical students before an anatomy exam, we have compared AS with expressive writing (EW). EW, or expressive disclosure of the thoughts and emotions about an upcoming exam, was shown to reduce the depressive symptoms in subjects who were taking stressful exams [12] and subsequently improved the exam performance in high school and university students [7,13].

Using the advantages of crossover design, as tested in previous trial [11], the present investigation compared AS with EW and no intervention in treatment of exam anxiety in medical students, who were randomly assigned to each of these 3 conditions. Based on above-mentioned data, we hypothesized, that both AS and expressive writing will reduce anxiety, improve sleep and thus enhance the academic performance of medical students during anatomy exams.

## Methods

### Study design and randomization

This prospective randomized controlled crossover trial was performed between April and July 2014 at the University of Greifswald, Germany. Crossover design means that each of the participants will receive all study interventions in a random order and thus will serve as her/his own control [14]. In comparison with parallel-arm design with its “between-participant” comparison, crossover design uses “within-participant” comparison, which enable statistically and clinically significant results with less participants than with a parallel-arm design [11,14].

The participants were recruited via announcement in March 2014 before the first anatomy exam in April 2014 according to the following eligibility criteria: undergraduate residential medical students in their first year of study with no fundamental knowledge about and experiences with acupuncture, undergoing three oral anatomy exams within three months, without local auricular skin infection, without history of alcohol abuse or use of opioid or psychotropic medication, and with an American Society of Anesthesiologists physical status score of I-II. None of the students were taking any medications or recreational drugs at the time of the study and all of them were paid (10 EUR/hour) for their participation. The investigation was finished on the day of the last anatomy exam in July 2014.

**Fig 1:**
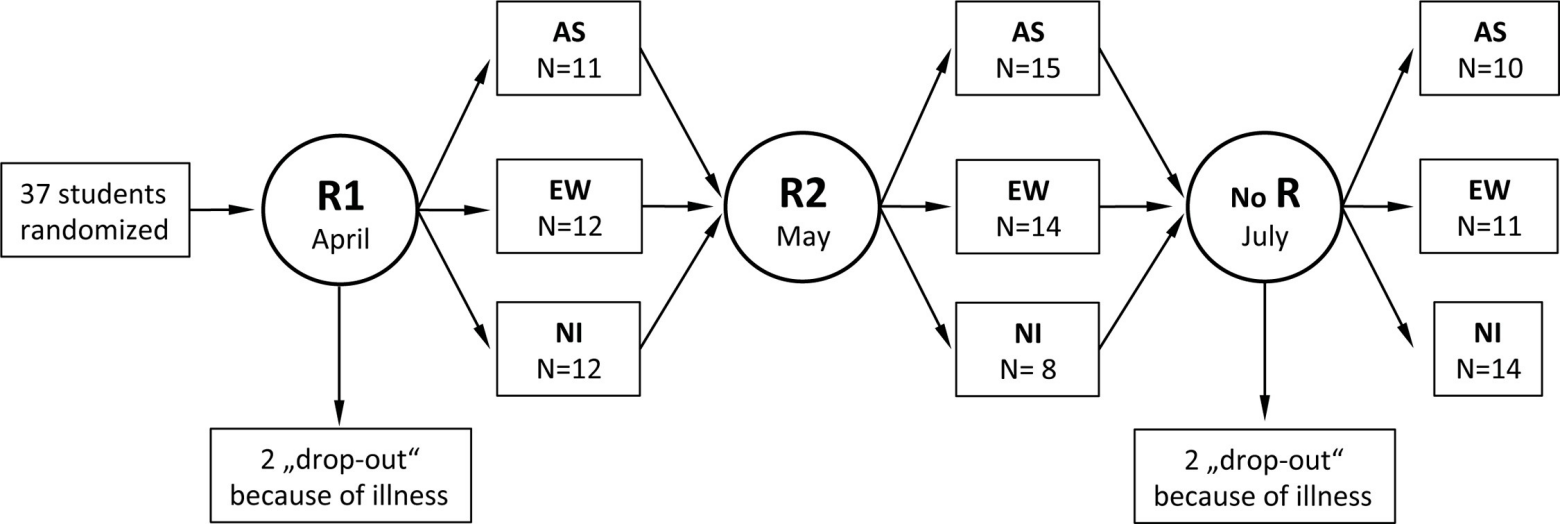
Flow of the investigation. First randomization (R1) was performed by drawing of wrapped pieces of paper with hidden numbers 1, 2 or 3 before the 1^st^ exam. Second randomization (R2) was performed before the 2^nd^ exam by flipping a coin. No further randomization was necessary before the last exam in July. R: randomization; AS: auricular acupuncture; EW: expressive writing; NI: no intervention condition.

The research project has been carried out in accordance with the Declaration of Helsinki, was approved by the Institutional Ethics Committee of the University Medicine of Greifswald on the 29^th^ of April 2014 (reference no. BB 037/14) and was registered at clinicaltrials.gov (registration number NCT03093584). This registration was performed later than the enrolment of the participants was started, since initially the authors regarded the project as an experimental investigation. The authors confirm that all ongoing and related trials for auricular stimulation for anxiety are registered at clinicaltrials.gov. The written informed consent was obtained from each participant after the nature of the study procedures was explained. As all students took three comparable anatomy exams with an interval of one month, each of them was randomly assigned to the AS, EW or no intervention condition at the evening prior to a scheduled exam by drawing slips of paper with the numbers 1, 2 or 3 out of a hat. Each number corresponded to an intervention, as determined a priori: 1 = AS, 2 = EW, 3 = no intervention before the first exam (Fig 1). Before the second exam, participants were randomly assigned to one of the two remaining conditions by flipping a coin. The investigator, who performed the randomization throughout the study, ensured that the participants could not have been randomized again to the condition they had before. Before the last exam, no further randomization was necessary. The investigator informed the acupuncturist about the assignment of the next participant immediately after the randomization procedure and prior to any intervention.

### Study interventions

#### Auricular stimulation

**Fig 2:**
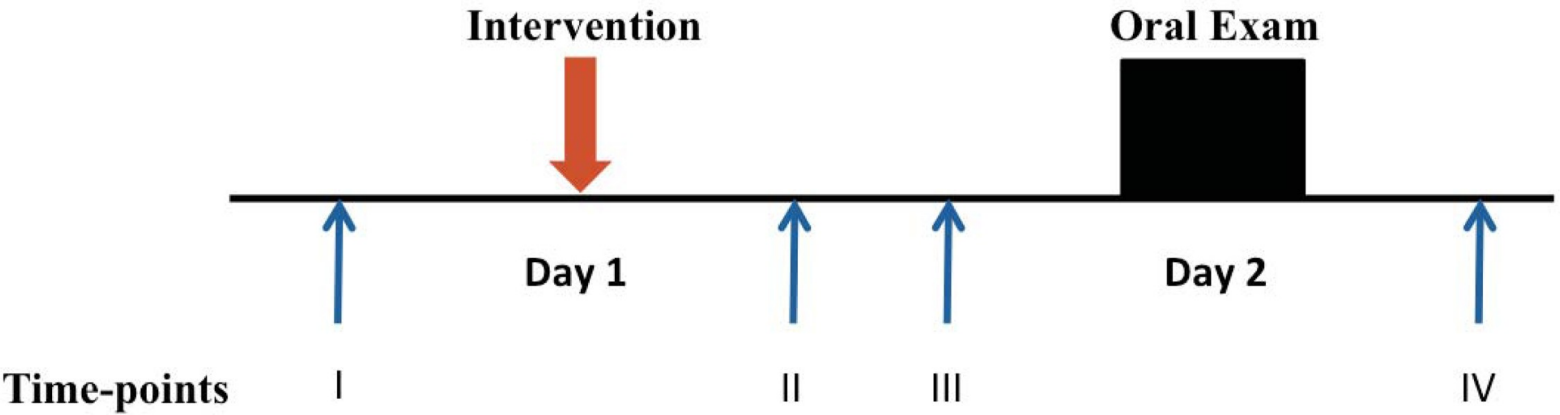
Timeline of study session. Time I: baseline; Time II: evening of the day before exam; Time III: immediately before the anatomy exam; Time IV: after exam. Anxiety was measured using the State-Trait-Anxiety Inventory (STAI) at Time I-III; salivary amylase was measured at Time II-IV; the quality of participants’ sleep one night before the exam was registered at Time III; hemodynamic values were registered at Time I, III and IV.

A licensed acupuncturist with more than five years of experience with this technique applied AS within 10 minutes at the acupuncture points MA-IC1 (Lung), MA-TF1 (ear Shenmen), MA-SC (Kidney), MA-AT1 (Subcortex) and MA-TG (Adrenal gland) bilaterally according to the methodology, which was previously described in detail elsewhere [15]. All these auricular acupuncture points are situated in the areas innervated by cranial nerves exclusively or receiving mixed innervation by the auricular branch of the vagal nerve (ABVN) and cervical nerves [8,16]. Indwelling fixed ‘New Pyonex’ needles (length 1.5 mm, diameter 0.22 mm; Seirin Corp, Shizuoka City, Japan) embedded in a skin-colored adhesive tape were used for AS. The participants were instructed by the acupuncturist to stimulate the auricular needles for 3–5 minutes, if they felt anxious before the exam. Auricular needles were left in situ until the next day and were removed immediately after the exam (Fig 2).

#### Expressive writing

For expressive writing, the standard protocol described by Lepore, 1997 [12], including the instructions translated in German (S2 File) was used. According to the method described [12], on the day before the anatomy exam the participants were asked to write their essays by hand on paper within 15–20 minutes in a separate comfortable office room with a low level of noise and lighting. Before writing, the verbal and written instruction (S2 File) was given to participants. Expressive writing was scheduled in a way that the participants were writing either alone or together with another person in the same room. After the intervention, the essays were collected and their content was analyzed using the German Version of the Linguistic Inquiry and Word Count test analysis program (LIWC2007, Pennebaker, Booth, & Francis, 2007). Based on previous studies on expressive writing [7,12,13], we chose the following linguistic categories as potentially related to writing-induced changes in anxiety and rumination: anxiety words (worried, fearful, nervous), affect words (happy, cried, abandoned), positive emotion words (love, nice, sweet), negative emotion words (hurt, ugly, nasty), inhibition words (block, constrain, stop), cause words (because, effect, hence), and insight words (think, know, consider). For analysis, two essays had to be excluded, because they were written in non-German languages.

#### No intervention

If the participants were assigned to the no intervention condition, they remained seated in the examination room for 10 to 15 min, which is the same amount of time an application of the AS needles or expressive writing procedure would have taken. During that time, the investigator conducted a conversation with the participants about leisure activities, place of birth, and opinions on the study facility, thereby, avoiding the topic of the upcoming exam.

### Outcome measures

Outcome measures were taken before the intervention (baseline, Time I, in the room, where the study interventions were applied); after the intervention in the evening of the day prior to exam (Time II, at participants’ home); immediately before (Time III, in the room in front of examination hall), and after the exam (Time IV, in the room in front of examination hall, Fig 2). The assessor of outcome measures was unaware regarding the participants’ allocation to study conditions. As the primary outcome, state (situational) anxiety levels were measured using the German version of Spielberger’s State-Trait-Anxiety Inventory (STAI; ranging from 20 (low anxiety) to 80 (highest level of anxiety); [17]) at Time I, II and III. In the morning of the exam (Time III) the participants were asked about the quality of sleep the night before: no change, better or worse than the quality of sleep during the previous week. Blood pressure and heart rate were measured before and after each intervention, as well as before and after each exam (Time I-IV, Fig 2). Saliva samples were taken at Time II, III and IV and the activity of salivary alpha-amylase (sAA) was measured subsequently using standardized spectrophotometry technique on the Dimension Vista 1500 (Siemens Healthcare GmbH, Eschborn, Germany). This assay utilizes a synthetic chromogenic substrate, 2-chloro-4-nitrophenol linked with maltotriose, which is hydrolyzed by sAA and the formed 2-chloro-4-nitrophenol is measured spectrophotometrically at 405 and 577 nm after incubation of 70 seconds at 37° C. The method responds to both pancreatic and salivary amylase isoenzymes. Samples were diluted prior to analysis and all measurements complied with the national legal requirements according to “German Medical Association on Quality Assurance in Medical Laboratory Examinations (Rili-BAEK)” [18]. After the exam, the test performance (pass or fail) was recorded.

### Statistical analysis

The sample size was calculated based on the mean difference of state anxiety levels (STAI) between conditions of 20 and a standard deviation of 20 from the previous investigation [15] by determining the two-sided level of significance at 0.01 (three-period crossover investigation) and power at 80% for a 2-tailed paired sample *t*-test. Expecting to find a 30% difference in anxiety levels between the different study conditions, the number of participants needed was calculated to be 24. Taking into account potential drop-out/withdrawal rate of 10–20%, the sample size was inflated to a total of 30 volunteers.

Baseline characteristics, as well as the differences between the study conditions at different time points, were analyzed using paired sample *t*-tests and Wilcoxon signed ranks test as appropriate, with subsequent Bonferroni adjustment for multiple comparisons. McNemar test was used to analyze the quality of sleep in the night before the anatomy exam. Changes of activity of sAA, blood pressure and heart rate in course of the study were analyzed using general linear model for repeated measures. The association of state anxiety with previously defined linguistic categories of LIWC2007 (see Expressive writing subsection above) was tested using a partial correlation with total word count as a covariate. Data analysis was performed using IBM SPSS Statistics Software for Mac (Version 19.0.0, IBM Corp., New York, USA). All data are presented as mean (standard deviation) unless otherwise stated; two-sided Bonferroni-adjusted *P*-values < 0.05 were regarded as significant.

## Results

### Baseline data

We have included 37 participants instead of 30, because additional 7 students, who previously had bad experience with exam anxiety and who knew about positive results of our previous investigation on treatment of exam anxiety in medical students one year ago [15], asked us to take part in this investigation. Thus, we have enrolled these 37 (25 females; mean age 23 ± 3) students, who all fit the inclusion criteria (Fig 1). Two female participants missed the first session and another two missed the third session because of illness (Fig 1), their data were treated as missing data.

### Anxiety

The trait anxiety, assessed with STAI was 46 ± 10, significantly exceeds the mean found in the norm sample for the female population aged between 15 and 29 years: 36 ± 10; *t*_37_ = 4.3; *P* < 0.001; d = 0.9 (15).

**Fig 3:**
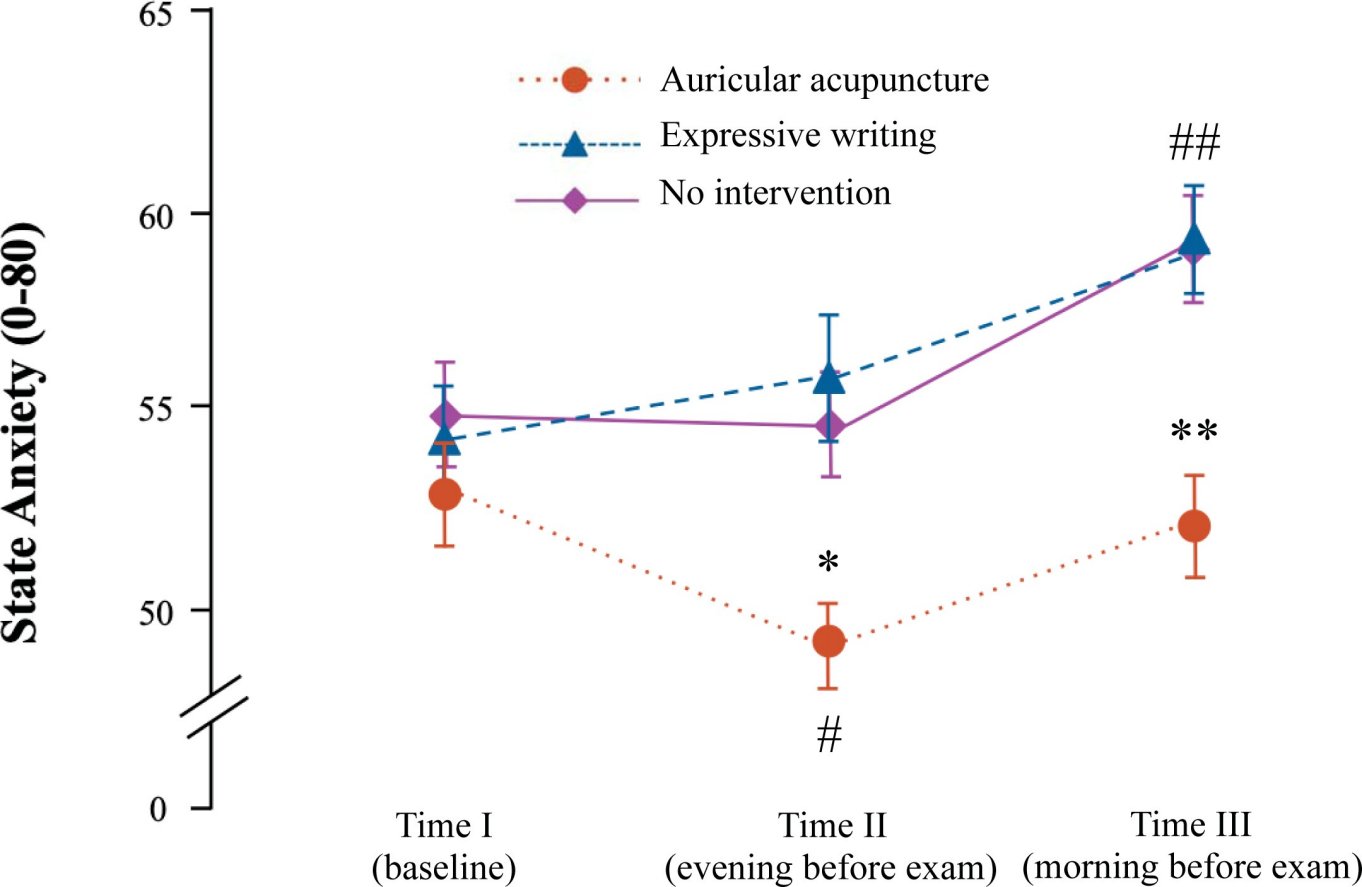
State exam anxiety measured using state-trait anxiety inventory. # means *P* = 0.015 for auricular stimulation (AS) at Time II vs. AS at baseline; ## means *P* < 0.01 for expressive writing (EW) and no intervention (NI) at Time III vs. EW and NI at baseline; * means *P* < 0.05 for AS vs. EW and vs. NI at time II; ** *P* < 0.001 for AS vs. EW and vs. NI at Time III; paired sample *t*-test; *P*-values Bonferroni adjusted; exact *P*-values are given in text. Data given as mean ± standard error of mean.

The baseline state anxiety levels, measured before intervention (Time I), were comparable among all three trial conditions. State anxiety levels measured 5 hours after expressive writing and after no intervention in the evening prior to the anatomy exam (Time II) remained comparable with baseline values (Table 1 and Fig 3).

After AS, state anxiety, measured 5 hours later, decreased in comparison with baseline values: mean difference (MD) = 4.0; 95% CI 1.2, 6.6; *t*_35_ = 3.0; *P* = 0.015; d = 0.5. State anxiety on the day of exam immediately before exam (Time III) was higher than at baseline after both expressive writing: MD = 5.0; 95% CI 2.1, 8.2; *t*_36_ = 3.4; *P* = 0.003; d = 0.5 and after no intervention condition: MD = 4.0; 95% CI 2.4, 6.8; *t*_32_ = 4.3; *P* < 0.001; d = 0.5 (Fig 3).

**Table 1:** Outcome measures of the investigation.

Parameter	Time of measurement	Intervention		
		EW	AS	No intervention
**State anxiety (Fig 3)**	I (baseline)	54.2 ± 9.2	53.3 ± 8.9	54.9 ± 9.0
	II (after intervention)	55.7 ± 10.7	49.1 ± 8.0	54.7 ± 8.7
	III (before exam)	59.4 ± 9.9	52.5 ± 9.5	59.1 ± 8.4
**Salivary amylase(U/ml) (Fig 5)**	II (after intervention)	205.1 ± 22.6	228.8 ± 35.9	187.7 ± 27.6
	III (before exam)	110.2 ± 16.8	147.6 ± 22.7	173.4 ± 32.0
	IV (after exam)	115.4 ± 19.1	141.3 ± 21.4	170.9 ± 27.1
**Passed exam, N (%)**	After exam	23 (62)	23 (62)	26 (70)

State anxiety presented as mean ±SD; amylase activity as mean ± SEM; EW: expressive writing; AS: auricular stimulation.

State anxiety level at Time II was lower after AS than after no intervention: MD = 5.6; 95% CI 1.6, 9.5; *t*_31_ = 2.9; *P* = 0.02; d = 0.5, as well as lower after AS than after expressive writing: MD = 6.3; 95% CI 2.5, 10.0; *t*_35_ = 3.4; *P* = 0.006; d = 0.6 (Fig 3). On the morning of the exam (Time III), state anxiety level after AS was also lower than after no intervention: MD = 5.9; 95% CI 3.3, 8.5; *t*_31_ = 4.7; *P* < 0.001; d = 0.8, and in comparison with expressive writing: MD = 6.5; 95% CI 3.4, 9.7; t_35_ = 4.2; *P* < 0.001; d = 0.7 (Fig 3).

### Expressive writing

The participants wrote essays with a mean word count of 297 ± 79 words (range 102–475, Table 2).

**Table 2:** Word count for the different LIWC categories in expressive writing (N = 35).

LIWC categories	Mean ± SD
**Anxiety**	1.33 ± 0.83
**Affect**	7.0 ± 1.76
**Positive Emotion**	3.16 ± 1.32
**Negative Emotion**	3.83 ± 1.56
**Inhibition**	0.27 ± 0.35
**Cause**	1.83 ± 1.03
**Insight**	4.12 ± 1.07

LIWC: Linguistic Inquiry and Word Count

Analysis of writing content (Table 2) using a partial correlation with total word count as a covariate showed that state anxiety levels at baseline (Time I) were positively correlated to the use of anxiety words (r = 0.34, P = 0.05) and negatively correlated to the use of positive emotion words (r = −0. 41, P < 0.05). At Time II and III state anxiety did not correlate with the use of words of any category, described in the Methods section.

### Quality of sleep

**Fig 4:**
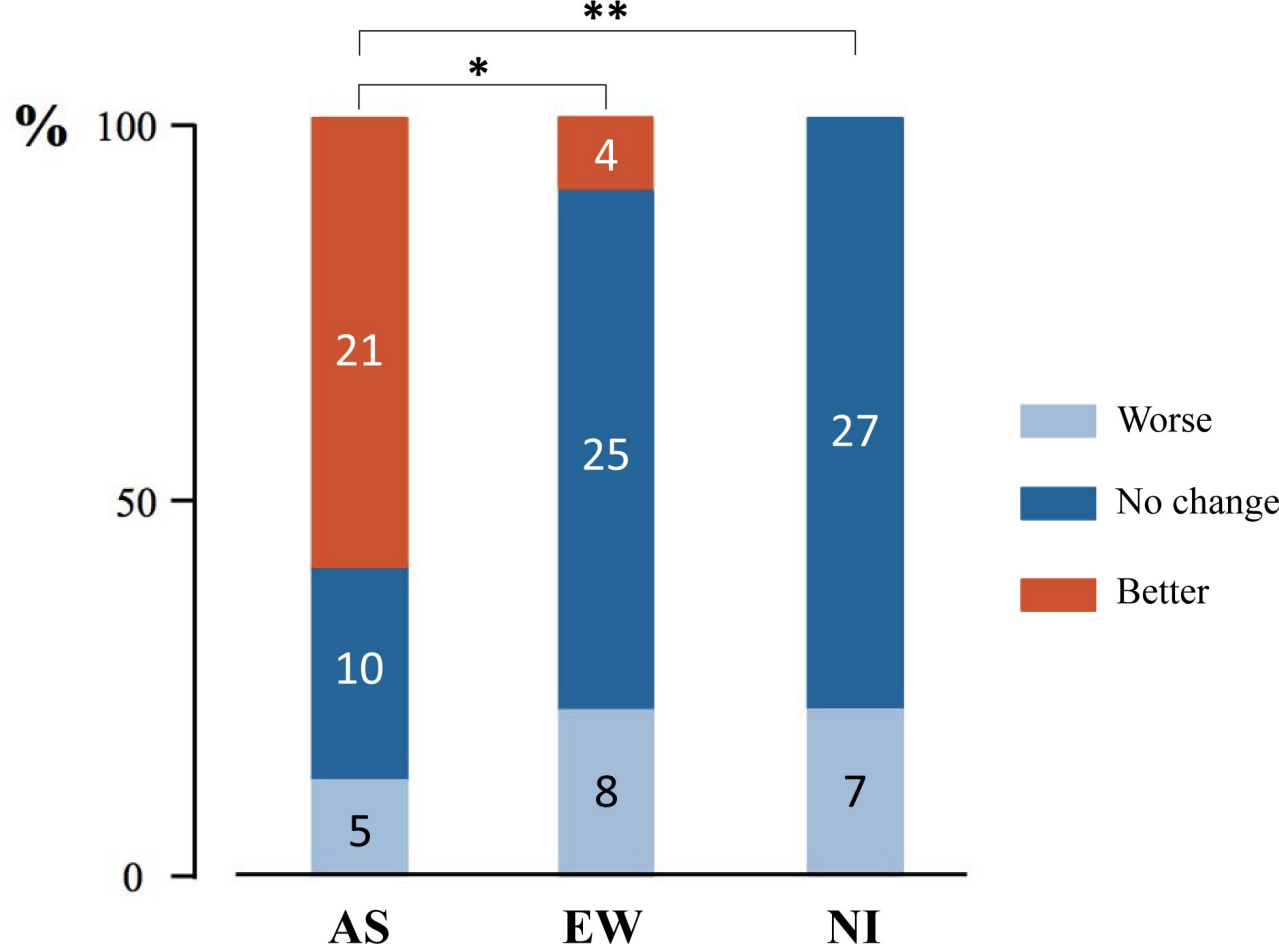
Quality of sleep in the night before exam given as per cent of participants. * *P* = 0.003 for comparison of AS vs. EW and ** for *P*< 0.001 for comparison of AS vs. NI; McNemar test; *P*-values Bonferroni adjusted. AS: auricular acupuncture; EW: expressive writing; NI: no intervention. Figures inside the bars are absolute numbers of participants.

Twenty-one participants reported better quality of sleep in the night before exam after AS in comparison with 4 participants after expressive writing (*P* = 0.003; Fig 4), whereas none of participants reported this improvement after no intervention (*P*< 0.001 for comparison of AS vs. no intervention).

### Salivary alpha-amylase

**Fig 5:**
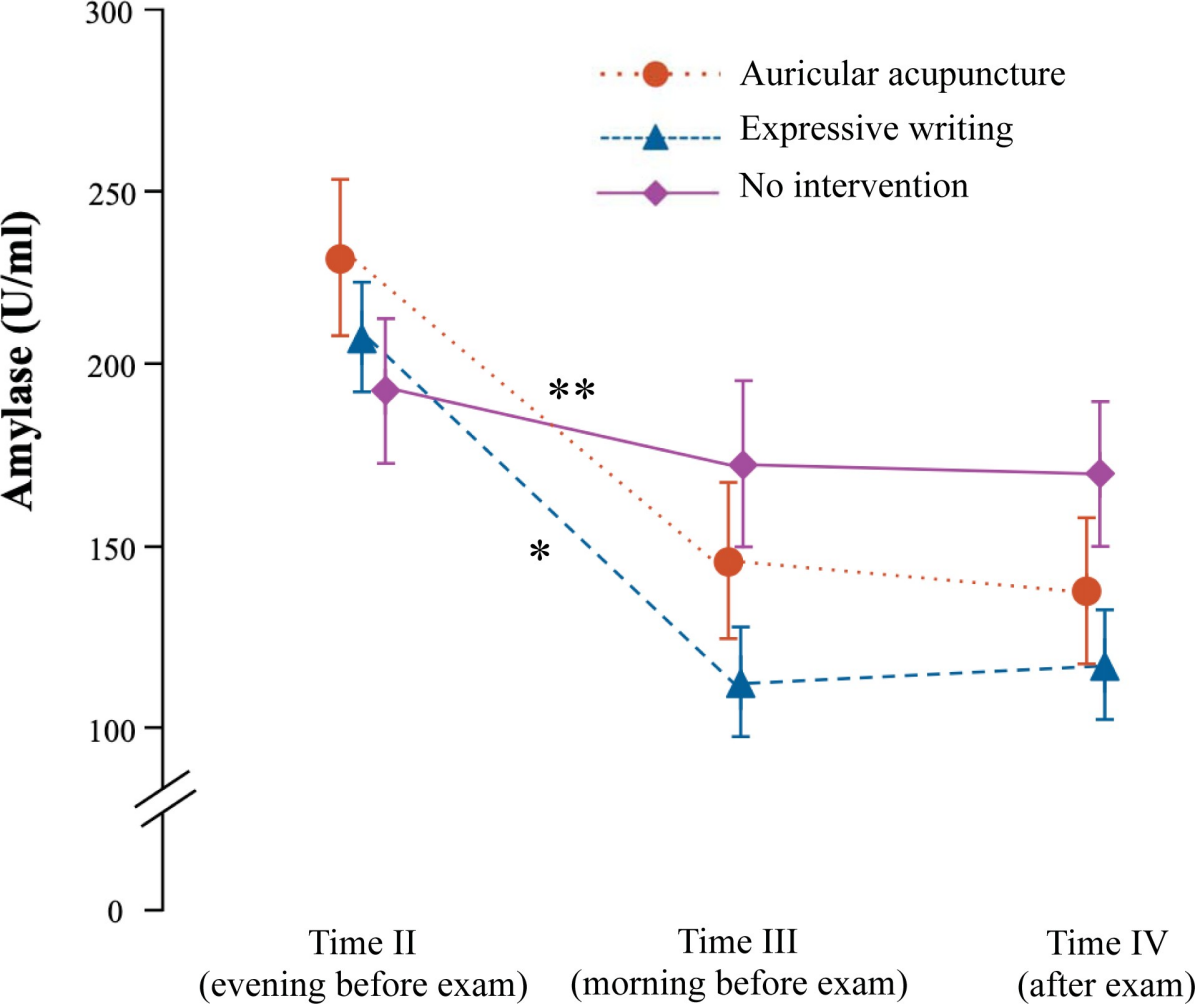
Activity of salivary alpha-amylase during the investigation. Time II: evening of the day before exam; time III: in the morning immediately before the anatomy exam; Time IV: after anatomy exam. * *P* = 0.001 for changes in time from baseline after expressive writing; ** *P* = 0.01 for changes in time from baseline after auricular acupuncture; general linear model for repeated measures. Data given as mean ± standard error of mean.

The levels of sAA activity were comparable among three trial conditions at all time-points of measurement (Table 1 and Fig 5). The activity of sAA was lower at Time III vs. Time II after expressive writing (F_2,66_ = 7.43; *P* = 0.001) and after AS (F_2,56_ = 5.02; *P* = 0.01) but not under no intervention condition (F_2,56_ = 0.38; *P* = 0.68).

### Hemodynamic parameters

**Fig 6:**
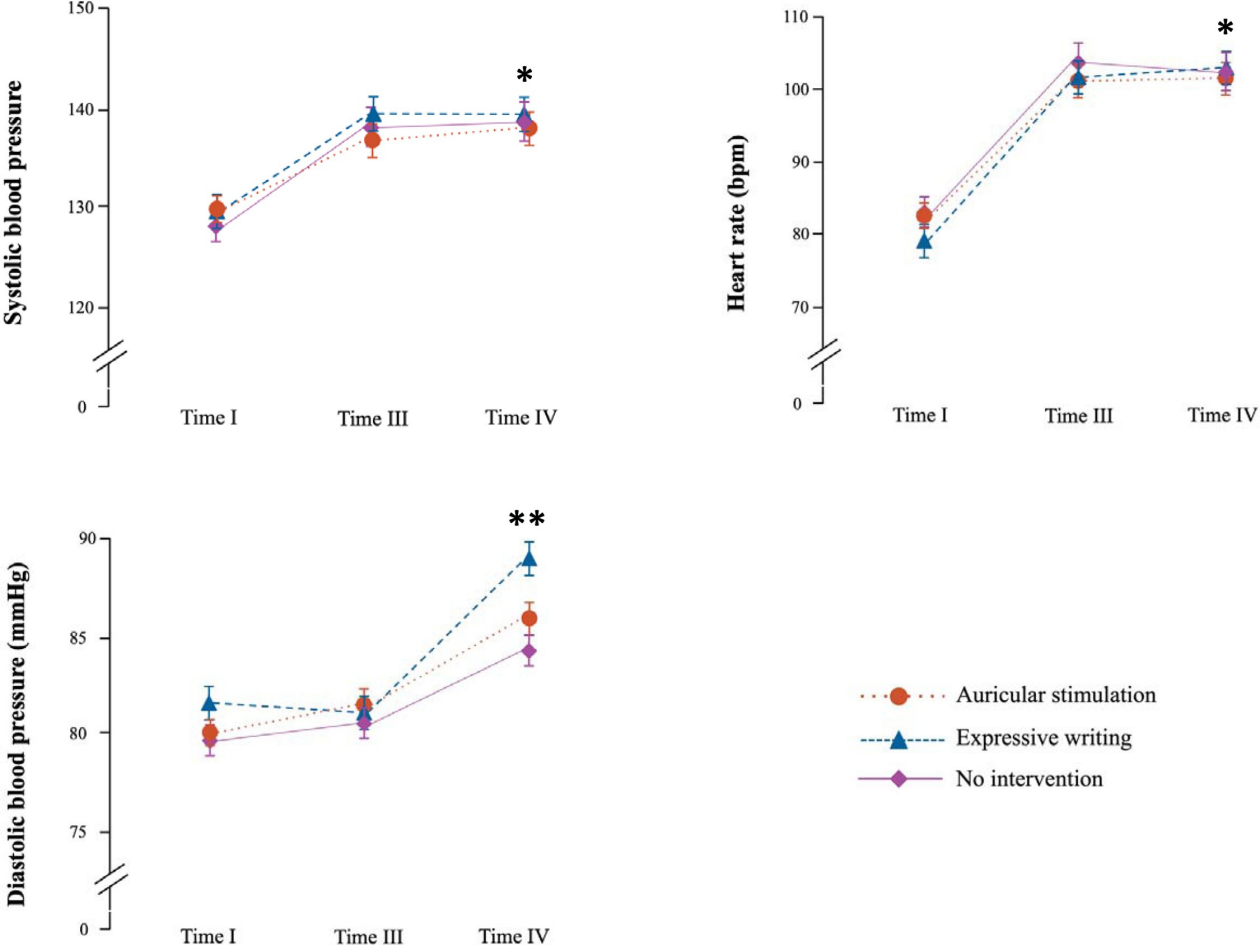
Hemodynamic parameters during the investigation. * *P*<0.01 for changes in time from baseline under all three study conditions. ** *P*<0.01 for changes in time from baseline under auricular acupuncture and expressive writing; general linear model for repeated measures. Data given as mean ± standard error of mean.

Systolic blood pressure and heart rate increased on the day of exam in comparison with baseline (*P*<0.01; Fig 6 and Supplementary Table) for all conditions. Diastolic pressure immediately after the exam was higher than baseline values after AS (F_2,70_ = 5.44; *P* = 0.006) and expressive writing (F_2,70_ = 7.96; *P* = 0.001), but not after no intervention (F_2,64_ = 2.41; *P* = 0.1).

### Exam performance

Exam performance was comparable among the three study conditions (Table 1).

## Discussion

In this crossover trial auricular “acupuncture-like” stimulation (AS) was better than expressive writing and no intervention conditions in the treatment of exam anxiety and improvement of sleep quality in medical students, without changing systolic blood pressure or heart rate. Expressive writing was comparable with no intervention condition in this regard.

The pattern of anxiety reaction in students under AS in the present investigation repeated the changes of anxiety levels, which were observed in the previous study [11]: state anxiety decreased after AS in comparison with baseline in the evening of the day prior to exam with comparable effect size and then returned to baseline level at the time-point immediately before exam (Fig 3). In contrast to our previous investigation, where the largest effect size of AS over control interventions was observed in the evening after AS on the day prior to the anatomy exam, the largest effect in present investigation (Cohen’s d = 0.7 and 0.8) was seen on the day of exam immediately before the exam. The effect size of AS after intervention vs. baseline observed in the present study is comparable with that from our previous investigations [11,15] and several other studies on the effects of AS on situational anxiety where the state anxiety decreased by about 15–20% from baseline after AS in patients scheduled for various medical procedures with high level of situational anxiety [9,10,19].

The no intervention condition in the present investigation yielded the results, which also repeat our previous data: the state anxiety increased constantly before the upcoming exam (Fig 3). These results are in agreement with the findings of Brockmeyer, who used a comparable design to assess the effect of AS and placebo procedures on situational anxiety before public speaking in university students [20].

Contrary to expectations based on previous reports about the effective use of expressive writing in students suffering from exam anxiety [7,12,13], in our investigation expressive writing had no effect on either state anxiety levels or exam performance. State anxiety level under the expressive writing condition increased from baseline through the day of exam and was indistinguishable from anxiety levels during the no intervention condition. The emotional content of expressive writing in our investigation (Table 2) was comparable to that from the essays of Ramirez & Beilock, 2011, suggesting the fair replication of their methodology [7]. However, previous investigations that favored expressive writing [7,12,13] did not study its effect on state anxiety levels, but rather explored the impact of expressive writing on depressive symptoms, particularly intrusive thoughts, and testing scores. The absence of the effect of expressive writing on exam performance in our study might be explained by the insensitivity of dichotomous values (“passed or not passed”), which were used for evaluation of anatomy exams.

The mean value of trait anxiety in this study group was comparable with the mean level of trait anxiety in students from our previous randomized trial on AS vs. placebo and no intervention in treatment of exam anxiety [11] and, as expected, was higher than the mean level found in the general female population aged between 15 and 29 years [17]. This is in agreement with previous findings about high levels of anxiety among undergraduate medical students before anatomy exams [21] and might have contributed to “natural” selection of predominantly anxious students who had volunteered to participate in our study.

In contrast to the results of our previous investigation [11], where we could not find the beneficial effect of AS or placebo on quality and duration of sleep, the present study did observe the improved sleep quality after AS. This finding is in agreement with the findings of Chueh et al (2018), who reported on improved sleep quality after auricular stimulation in anxious nurse students [22].

The activity of salivary alpha-amylase (sAA) was lower before anatomy exam in comparison with the evening before exam after both AS and expressive writing, but not after the no intervention condition. Since sAA is considered a surrogate non-invasive marker of sympathetic nervous system activity [23], this finding might be explained as anxiolytic effect of both auricular stimulation of cranial nerves and expressive writing interventions, both of them able to influence the autonomic nervous system. Unfortunately, the lack of baseline activity of sAA in stress-free conditions precludes the definitive conclusion. Mean values of sAA level after AS before and after anatomy exam were higher than expressive writing and lower than no intervention, however this finding might be accidental due to small sample of present investigation.

The reaction of hemodynamic parameters—constant increase from baseline to exam–is in agreement with previous findings [4,24]. After the exam, only diastolic pressure increased further, whereas systolic blood pressure and heart rate remained unchanged as compared to measurement immediately before the exam [24].

This trial followed the recommendations of the experts for conducting and reporting of acupuncture studies [25]. We believe that due to crossover design and use of a constant pattern of cranial nerve stimulation rather than individualized acupuncture, the potential biases were minimal. The dropout rate of 11% in this investigation was low.

However, despite the fact that calculated sample size was sufficient for the present experimental crossover investigation, this small sample and the prevalence of female participants in the study preclude the generalizability of the findings over the populations. Although we believe, that 3-period crossover design of our investigation has eliminated the potential influence of female reproductive cycle on anxiety [26], we did not control for this bias factor in our study. Moreover, a larger sample size is necessary to enable the normal distribution of sAA values in order to achieve the reliable laboratory findings in future investigations. Also, the number of participants who stimulated the needles by pressing, if they felt anxious, should be verified, since this might have biased the results of our investigation. Regarding the above-mentioned limitations of the present investigation, we suggest that future studies should examine larger samples with improved methodology and compare AS with methods commonly used for treatment of exam anxiety, e.g. relaxation techniques and biofeedback [5,27,28]. Moreover, after appropriate investigations, AS using present methodology might be used to treat pre-operative anxiety in surgical patients [9,10,19,29].

## Conclusion

Auricular acupuncture-like stimulation, applied to the ear areas innervated mostly by the auricular branch of the vagal nerve, reduced exam anxiety and improved quality of sleep in medical students as compared with expressive writing and no intervention conditions. These changes might be due to reduced activity of sympathetic nervous system.

## Supporting information

S1 File CONSORT checklist. (DOCX)

S2 File Instructions for expressive writing. (DOCX)

S3 File Study protocol. (PDF)

S4 File Raw data. (SAV)
