# Supplementary material for: Auricular stimulation vs. expressive writing for exam anxiety in medical students – A randomized crossover investigation
Source: PLoS One. 2020 Aug 27;15(8):e0238307. doi: 10.1371/journal.pone.0238307 (PMC7451547; doi:10.1371/journal.pone.0238307)
Supplement: S2 File — (DOCX) [file pone.0238307.s002.docx]

Appendix A

**Instructions for writing the essay**

We beg you to deal seriously with the topic “Exam Anxiety”. Please read your task, written below, attentively. You have around 15-20 minutes to complete the essay.

Essay Expressive Writing

During todays session of writing please, try to relax yourself and try to describe your most deep thoughts and feelings about your exam, which will take place tomorrow.

In your essay you can express yourself about your thoughts and feelings, about exam itself, about the impact of the exam on your everyday life. Also please disclose your ideas and emotions concerning the consequences of the exam note on your future plans and may be your alternative plans, which you may already have. It is extremely important that you express your deepest emotions regarding the exam and record them within this essay.

*Original instruction (Lepore et al., 1997)*

*During today's writing session, I want you to let go and write about your very deepest thoughts and feelings about the exam. In your essay, you may want to write about your thoughts and feelings regarding the exam itself, the effect of the exam on your life in the present, the exam's implications for your future goals, and alternate plans you may have. The important thing is that you dig down into your deepest emotions and explore them in your writing.*
