# Supplementary material for: Auricular stimulation vs. expressive writing for exam anxiety in medical students – A randomized crossover investigation
Source: PLoS One. 2020 Aug 27;15(8):e0238307. doi: 10.1371/journal.pone.0238307 (PMC7451547; doi:10.1371/journal.pone.0238307)
Supplement: S3 File — (PDF) [file pone.0238307.s003.pdf]

**Titel:** Auricular acupuncture (AA) vs. expressive writing in treatment of pre-exam anxiety - randomised crossover study in medical students

04/2014 – 07/2014

**Geplante Dauer:**

**Leiter der Studie:** PD Dr. T. Usichenko
Klinik für Anästhesiologie und
Intensivmedizin
Universitätsmedizin Greifswald
Fleischmannstraße 42-44
D-17475 Greifswald
Telefon: 049-(03834) 865893
Telefax: 049-(03834) 865802
E-Mail: taras@uni-greifswald.de

# Contents

| | | |
|---|---|---|
| | CONTENTS | 2 |
| 1. | GENERAL INFORMATION | 3 |
| 1.1 | Institution and investigators | 3 |
| 1.2 | Synopsis | 4 |
| 2. | SCIENTIFIC BACKGROUND AND AIMS OF THE STUDY | 5 |
| 2.1 | Background | 5 |
| 2.2 | Aims of the study | 6 |
| 3. | ETHICAL ASPECTS | 6 |
| 3.1 | Benefit-risk considerations | 7 |
| 3.2 | Protection of patients | 7 |
| 4. | STUDY DESIGN | 7 |
| 5. | PATIENTS' SELECTION | 8 |
| 5.1 | Inclusion criteria | 8 |
| 5.2 | Exclusion criteria | 8 |
| 5.3 | Discontinuation criteria | 8 |
| 6. | TREATMENT PLAN | 9 |
| 6.1 | Group allocation | 9 |
| 6.2 | Study procedure | 9 |
| 6.3 | Outcome measures | 10 |
| 7. | SAMPLE SIZE AND STATISTICS | 10 |
| 8. | REFERENCES | 11 |
| 9. | APPENDICES | |
| | Appendix A. Poster Klausenitz et al. | 12 |
| | Appendix B. Einverständniserklärung | 13 |
| | Appendix C. Probandeninformation über die Studie | 14 |
| | Appendix D. Anleitung "Expressives Schreiben" | 18 |
| | Appendix E. Studienprotokoll | 19 |

# 1. GENERAL INFORMATION

## 1.1. Institutions and Investigators

**Institutions:** Klinik für Anästhesiologie und Intensivmedizin
Universitätsmedizin Greifswald
Fleischmannstraße 42-44
D-17487 Greifswald
Telefon: 049-(03834) 865893
Telefax: 049-(03834) 865802

**Principal Investigator:** PD Dr. Taras Usichenko
Klinik für Anästhesiologie und Intensivmedizin
Universitätsmedizin Greifswald

**Investigators on site:** Anna Wenzel
Catharina Klausenitz
Studentinnen Humanmedizin
Universitätsmedizin Greifswald

## 1.2. Synopsis

**Title of the study:** Auricular acupuncture (AA) vs. expressive writing for pre-exam anxiety - a randomised crossover study

**Study period:** 04 / 2014 – 07 / 2014

**Principal Investigator:** PD Dr. T. Usichenko
Department of Anaesthesiology and Intensive Care Medicine
University Medicine of Greifswald

**Aim of the study:** To investigate the anxiolytic effect of AA vs. expressive writing and vs. no intervention in students, passing the oral exams in anatomy at the University of Greifswald

**Design:** Prospective randomised crossover trial

**Interventions:**
1. AA using indwelling fixed needles, retained 24 h *in situ*
2. Expressive writing

**Number of volunteers:** N = 30

**Inclusion criteria:**
Healthy medical students at the University of Greifswald
Participants of the anatomy exams in spring/summer 2014
Without previous anxiolytic, sedative and analgesic medication
No pregnancy or lactating
Informed consent

**Outcome measures:**
Anxiety level
Heart rate, blood pressure
Salivary α-amylase

# 2. SCIENTIFIC BACKGROUND AND AIMS OF THE STUDY

## 2.1 Background

### 2.1.1 Pre-exam anxiety

Pre-exam anxiety is widely spread among students (1,2) and influences both their physical and psychological state before the exams (3,4). Moreover, recent investigations demonstrated, that high levels of pre-exam anxiety can influence the exam scores (5-7).

### 2.1.2 Auricular acupuncture for pre-exam anxiety

Several interventions are known to reduce the fear. Writing about testing worries and cognitive behavioural therapy are the most common effective methods described previously (6,7). Concerning the auricular acupuncture (AA), it was shown that this intervention is effective in reducing clinical anxiety, such as pre-surgery (8) and pre-dental treatment anxiety (9).

Two years ago we studied whether AA applied to specific auricular acupuncture points is better than placebo AA and no intervention for treatment of pre-exam anxiety (Ethikantrag Nr. BB 49/12 25. April 2012, reference ). We enrolled healthy medical students from our University according to eligibility criteria. Each student, who anyway underwent 3 comparable exams with an interval of 1 month, received either verum AA, placebo or no intervention (“waiting list”); the order of interventions was randomised. AA was applied using indwelling fixed needles bilaterally on the day before exam. “New Pyonex” placebo needles were used for control procedure. Anxiety level, measured using visual analogue scale 100 mm (VAS-100) and State-Trait-Anxiety Inventory (STAI), duration of night sleep, blood pressure, heart rate, salivary cortisol and the quality of participants’ blinding were measured and analysed. Anxiety levels (both VAS-100 and STAI) were reduced after verum and placebo interventions compared to baseline and waiting list ($p<0.01$), verum AA was better than placebo immediately after AA ($p=0.03$). Duration of sleep also improved after both interventions ($p<0.001$) with no difference between verum and placebo (**Appendix A**). Thus, both verum and placebo auricular acupuncture reduced pre-exam anxiety and duration of sleep in medical students, whereas verum acupuncture was better than placebo acupuncture in treatment of anxiety (10).

In our present investigation we want to know the genuine “clinical effectiveness” of AA in treatment of pre-exam anxiety in medical students before anatomy exam. That is why we are going to perform the study, comparing the effectiveness of verum AA with standard psychological method “expressive writing” (6). Expressive writing, or expressive disclosure of the thoughts and emotions about upcoming exam, was shown to reduce the depressive symptoms in subjects who were taking stressful exams (11) and subsequently improved the exam performance in high school and university students (6,12).

## 2.2 Aims of the Study

Primary objective:
To compare the anxiolytic effect of AA vs. expressive writing and no intervention using Visual Analogue Scale 100 mm to measure the pre-exam anxiety.

Secondary objectives:
To investigate the influence of AA vs. expressive writing and no intervention on anxiety scores, blood pressure, heart rate and salivary α-amylase, as the measures of pre-exam stress reaction under 3 interventions during the study.

# 3. ETHICAL ASPECTS

The present study protocol complies with the requirements of the following directives and guidelines:

- Declaration of Helsinki (48 General Assembly, Somerset West, South Africa, 1996)
- Good Clinical Practice in the Conduct of Clinical Trials on Medicinal Products for Human Use (Brussels, 20.06.96, E6GCPD12/01 May 96)
- Berufsordnung für die deutschen Ärzte (Dtsch Ärzteblatt 94, 2354 - 2363, 1997)
- Standards of the Department of Anesthesiology and Intensive Care Medicine, Ernst Moritz Arndt University of Greifswald

## 3.1 Benefit-risk consideration

The patients receiving both AA and expressive writing about exam are expected to have benefit in reduction of anxiety intensity; thus the improved exam performance is hypothesized. The prognosis for the beneficial effect of AA is based on the results of randomized controlled studies (8,9) and our experience from the previous investigation using the same intervention (10).

The most common side effects of AA, described in case reports, were endocarditis and perichondritis (13,14). Therefore, we shall not include the subjects at risk (with damaged or prosthetic cardiac valves) for endocarditis from our investigation. We did not observe serious side effects of AA in more than 600 patients treated with indwelling fixed needles during the prospective clinical trials on analgesic effects of AA.

## 3.2 Protection of Participants

Ethics Committee

The protocol including the copies of the “Informed Consent Form” (**Appendix B**) and ”Special Information for Participants” (**Appendix C**) will be submitted to the Ethics Committee of the University of Greifswald. Written approval of the study protocol and the proposed “Informed Consent Form” and “Special Information for Participants” should be obtained before the start of the study.

Participant information

The participants will be informed both orally and in a written form about the study procedure, potential benefits and risks of the participation in the study, the right to withdraw from the study at any time, and to obtain written informed consent prior to any study procedures.

Insurance

The question about participants’ insurance (any harms and injuries, caused by the study or during the study) will be clarified with Allianz Insurance Company.

# 4. STUDY DESIGN

This will be a prospective blinded randomized crossover study at one centre – the University of Greifswald. The total duration of the study will be six days for each volunteer. These 6

days are divided in three periods, two days each, because of three anatomy exams (1 x in spring and 2 x in summer). Two days of investigation are necessary for each exam. The ethics committee should approve the design of the study. A total of 30 healthy volunteers scheduled for the oral exams in anatomy will be recruited according to participants' selection criteria.

# 5. PARTICIPANTS' SELECTION

## 5.1 Inclusion Criteria

1. Medical students at the University of Greifswald
2. Going to take part in oral exams of human anatomy
3. Participants without previous anxiolytic medication
4. Ability to express the thoughts and emotions ("expressive writing")
5. Written informed consent

## 5.2 Exclusion Criteria

1. Recidivist alcoholics
2. Local auricular skin infection
3. Pregnant or lactating women
4. Participants with prosthetic or damaged cardiac valves, intracardiac and intravascular shunts, hypertrophic cardiomyopathy and mitral valve prolaps (risk of bacterial endocarditis according to guidelines of AHA)
5. Participants who are unable to understand the consent form
6. History of psychiatric disease

## 5.3 Discontinuation Criteria

1. In case of intercurrent disease
3. All participants are free to withdraw from participation in this study at any time, for any reason, without penalty or loss of benefits.

# 6. TREATMENT PLAN

## 6.1 Group Allocation

Following the signing of the informed consent for participation in this study (**Appendix B**) along with the detailed participants' information about the study interventions for reducing pre-exam anxiety (**Appendix C**), all study participants will be consecutively randomised to receive either AA, expressive writing or no intervention on the day before anatomy exam. The randomisation before the first period of this 3-period crossover investigation will be performed using a list of closed envelopes, containing 3 conditions for the allocation (AA, expressive writing or no intervention). The randomisation before the second period of the investigation will be performed tossing a coin.

## 6.2 Study procedure

Five ear acupuncture points MA-IC1, MA-TF1, MA-SC, MA-AH7 and MA-T will be needled bilaterally in students during AA condition (**Appendix A)**. The choice of the ear acupuncture points is based on our previous investigation (10). Disposable indwelling steel ear acupuncture needles "New Pyonex" Seirin Corp. (Japan), sized 0.22 x 1.5 mm, will be used for this procedure.

The needles will be placed on the day before the exam, fixed with the skin-colored adhesive tape and retained in the ear until the following day after the exam. Before the needle insertion, the ears should be completely disinfected with alcohol swabs. For expressive writing, the standard protocol described by Lepore, 1997 (11), including the instructions translated in German (**Appendix D**), will be used. According to the method described (11), on the day before exam the participants are asked to write their essays within 15-20 minutes in a separate comfortable laboratory room with a low level of lighting. Before writing, the verbal and written instruction will be given to participants (**Appendix D**).

Physiological parameters, such as blood pressure, heart rate, are measured before and after the insertion of the needles/expressive writing as well as before the exam (**Appendix E**). In addition the salivary samples will be collected on the day before the exam after intervention, on the day of exam before and after the exam as well as on the control without any

psychological stress. The activity of enzyme α-amylase, as an equivalent of psychological stress, will be measured in the obtained salivary samples.

Anxiety scores, measured using STAI (State-Trait-Anxiety Inventory) and the VAS (Visual Anxiety Scale) will be filled out by the participants before and after the insertion of the acupuncture needles as well as before the exam. The questionnaires about the duration and quality of sleep will be filled out on the morning following acupuncture treatment.

The needles will be retained *in situ* until the day of the exam. In case of local inflammation or any adverse effects, potentially connected with ear needling, acupuncture needles will be immediately removed and participants will be withdrawn from the study.

### 6.3 Outcome Measures

Primary outcome measure: level of anxiety taken using Visual Anxiety Scale-100 mm

Secondary outcome measures:
-State and trait anxiety, using validated STAI (State-Trait-Anxiety Inventory)
-duration and quality of sleep
-physiologic parameters–heart rate, blood pressure, salivary α-amylase
-the quality of volunteers' blinding (volunteers' opinion about the group allocation)

## 7. SAMPLE SIZE AND STATISTICS

In order to calculate the appropriate sample size we set the level of significance to 0.01 (crossover investigation with multiple comparisons) and power at 80%. Taking the mean and standard deviation values of anxiety level, measured using VAS-100 mm in our previous investigation (10) and using the online sample size calculator (16) the number of volunteers was calculated to be 24 per condition. Taking into account potential drop-out/withdrawal rate of 10-20%, the sample size was set to a total of 30 volunteers. Normally distributed continuous data will be compared using the Student's *t*-test and an analysis of variance for paired observations, as appropriate. Skewed data will be compared using Friedman. Chi-square test will be used to analyse the success of volunteers' blinding and the incidence of side effects. SPSS Statistics Software for Mac (Version 21.0) will be used for data analysis.

# 8. REFERENCES


1. Holm-Hadulla R, Hofmann FH, Sperth M, Funke J: Psychische Beschwerden und Störungen von Studierenden. *Psychotherapeut* 2009; 54: 346–56.
2. Eisenberg D, Hunt J, Speer N: Mental health in American colleges and universities. *J Nerv Ment Dis* 2013; 201: 60–7.
3. Zhang Z, Su H, Peng Q, Yang Q, Cheng X. Exam anxiety induces significant blood pressure and heart rate increase in college students. *Clin Exp Hypertens* 2011;33:281-6.
4. Latas M, Pantic M, Obradovic D. Analysis of test anxiety in medical students. *Med Pregl* 2010;63:863-6.
5. Conley C, Travers L, Bryant F: Promoting psychosocial adjustment and stress management in first-year college students. *J Am Coll Health* 2013; 61: 75–86.
6. Ramirez G, Beilock SL. Writing about testing worries boosts exam performance in the classroom. *Science* 2011;331(6014):211-3.
7. Orbach G, Lindsay S, Grey S. A randomised placebo-controlled trial of a self-help Internet-based intervention for test anxiety. *Behav Res Ther* 2007;45:483-96.
8. Wang SM, Peloquin C, Kain ZN. The use of auricular acupuncture to reduce preoperative anxiety. *Anesth Analg* 2001;93:1178-80.
9. Karst M, Winterhalter M, Münte S, Francki B, Hondronikos A, Eckardt A, Hoy L, Buhck H, Bernateck M, Fink M. Auricular acupuncture for dental anxiety: a randomized controlled trial. *Anesth Analg* 2007;104:295-300.
10. Klausenitz C, Leutzow B, Kohlmann T, Wendt M, Usichenko TI. Auricular acupuncture for pre-exam anxiety in medical students - a randomised controlled trial. iSAMS Conference, Karolinska Institutet, Stockholm 2013.
11. Lepore SJ. Expressive writing moderates the relation between intrusive thoughts and depressive symptoms. *J Pers Soc Psychol* 1997;73:1030-7.
12. Frattaroli J, Thomas M, Lyubomirsky S. Opening up in the classroom: effects of expressive writing on graduate school entrance exam performance. *Emotion* 2011;11:691-6.
13. Rampes H. Adverse reactions to acupuncture. In: Medical acupuncture-a western scientific approach. Ed: Filshie J, White A. Chirchill livingstone, 1998.
14. Lee and McIlwain Subacute bacterial endocarditis following ear acupuncture Int J Cardiol 1985;7:62-3.
15. Lewis et al. Alternative Aproach to Premedication: Comparing Diazepam with Auriculotherapie and a Relaxation Method,1987
16. http://www.stat.ubc.ca/~rollin/stats/ssize

# APPENDICES

## Appendix A

# Auricular acupuncture for pre-exam anxiety in medical students – a randomised controlled trial

KLAUSENITZ, Catharina[1]; LEUTZOW, Bianca [1]; KOHLMANN, Thomas [2]; WENDT, Michael[1], USICHENKO, T

[1] Department of Anaesthesiology and Intensive Care Medicine; [2] Institute of Community Medicine; University Medicine of Greifswald

isams 2013

## OBJECTIVE

Auricular acupuncture (AA) is used to treat situational anxiety in clinical setting (1,2). We studied whether AA can reduce pre-exam anxiety vs. placebo and "waiting list" in students.

## METHODS

### PARTICIPANTS

- Healthy medical students undergoing oral anatomy exams
- Enrolled according to eligibility criteria
- Naive to AA
- Signed informed consent

### FLOW OF THE TRIAL

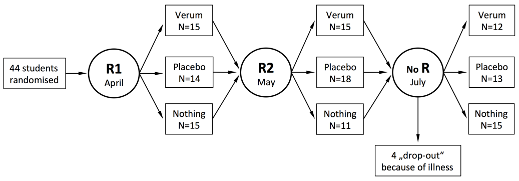


**Fig. 1.** Flow of the trial: * in July no further randomisation was necessary.

### INTERVENTIONS

- Verum AA: 0.22 x 1.5 mm indwelling fixed needles inserted at MA-IC1, MA-TF1, MA-SC, MA-AH7 & MA-T bilaterally
- "New Pyonex" placebo needles attached to the helix of the ear (Fig. 2)

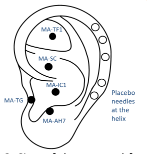


**Fig. 2.** Sites of the ear used for verum (black) and placebo (white circles) interventions.

### ENDPOINTS*

- Anxiety level measured using Visual Analogue Scale 100 mm (VAS-100) and State-Trait-Anxiety Inventory (STAI)
- Duration of night sleep and salivary cortisol
- Heart rate and blood pressure
- Quality of participants' blinding

* The endpoints of this study were fixed and sample size calculated after a pilot investigation

### FLOW OF THE STUDY SESSION

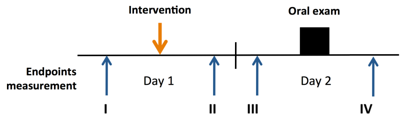


**Fig. 3.** Anxiety levels were measured using VAS-100 and STAI questionnaire at time-points I-III; salivary cortisol was measured in the morning before exam, hemodynamic values were registered at time-points I-IV; the quality of participants blinding was registered after exam.

## RESULTS

- Thirty-five participants were females, the age of the students was 23 ± 3 (mean ± SD) years
- Forty-four included participants finished 2 sessions, 4 of them missed the 3rd session because of illness
- Anxiety levels (both VAS-100 and STAI) were reduced after verum and placebo interventions compared to baseline and waiting list (ANOVA with repeated measures; p<0.01)
- Verum AA was better than placebo immediately after intervention on the day 1 (ANOVA with Bonferroni adjustment; p=0.03; Fig. 4A)
- Duration of sleep improved after both interventions, but this difference did not reach statistical significance (Fig. 4B).

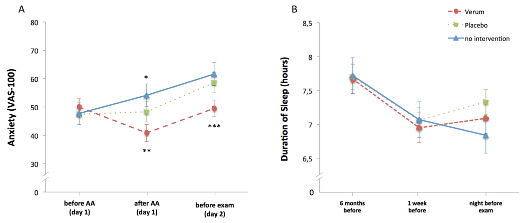


**Fig. 4. A.** Anxiety levels were measured using VAS-100 through the course of the study. *means p=0.003 for comparison verum AA vs. no intervention; **means p=0.03 for verum AA vs. placebo; ***means verum AA vs. no intervention. **B.** Duration of sleep 6 months, 1 week and one night before the anatomy exam; no statistically significant differences were found among the conditions. Data are given as mean ± SEM; analysis was performed using ANOVA with Bonferroni adjustment.

- There were no differences among the conditions in hemodynamic parameters and salivary cortisol levels
- Participants could distinguish between verum and placebo intervention (Fischer's exact test; p<0.001; Table 1)

**Table 1. Participants' opinion about group allocation**

| | After verum AA (N=41) | After Placebo (N=36) |
|---|---|---|
| It was verum | 34 | 7 |
| It was placebo | 6 | 21 |
| Don't know | 1 | 8 |

## CONCLUSION

Both verum and placebo AA reduced pre-exam anxiety in medical students. The superiority of verum over placebo might be due to insufficient participants' blinding.

## REFERENCES

1 Karst et al. Auricular Acupuncture for Dental Anxiety: A Randomized Controlled Trial. *Anesth Analg* 2007;104:295-300.

2 Michalek-Sauberer et al. Auricular acupuncture effectively reduces state anxiety before dental treatment—a randomised controlled trial. *Clin Oral Investig* 2012;16:1517-22.

## Appendix B

### Untersuchung zu Ohrakupunktur im Vergleich mit Expressivem Schreiben gegen Prüfungsangst bei Medizinstudenten

Name, Vorname des Probanden:

Geburtsdatum:

Ich wurde von dem unterzeichnenden Arzt über Art, Ziel und Ablauf der Studie und die zu erwartenden Wirkungen und Risiken aufgeklärt und habe die spezielle Probandeninformation gelesen und verstanden. Ich hatte auch ausreichend Gelegenheit, Fragen zu stellen und habe momentan keine weiteren Fragen.

Meine Teilnahme an der Studie ist freiwillig und ich kann jederzeit, auch ohne Angabe von Gründen, die Teilnahme an der Studie beenden, ohne dass mir dadurch Nachteile entstehen. Während meiner Teilnahme werde ich mich an die Anordnungen der Ärzte halten.

Ich bin damit einverstanden, dass die im Rahmen der Studie erhobenen Daten zur wissenschaftlichen Auswertung in anonymer Form verwendet werden dürfen. Weiterhin bin ich damit einverstanden, dass meine personengebundenen Daten zur Qualitätskontrolle und Qualitätsüberwachung der klinischen Prüfung durch die interne Qualitätssicherung der Klinik für Anästhesiologie und Intensivmedizin und die zuständige Überwachungsbehörde eingesehen werden dürfen.

Ich erkläre mich bereit, zu den obigen Bedingungen an dieser Prüfung teilzunehmen. Ich unterliege derzeit keiner Sperrfrist durch ein anderes Institut.

Greifswald, den Unterschrift des Probanden:

Greifswald, den Unterschrift des aufklärenden Arztes:

## Appendix C

**Untersuchung zu Ohrakupunktur im Vergleich mit Expressivem Schreiben gegen Prüfungsangst bei Medizinstudenten**

Kurztitel: Ohrakupunktur, Expressives Schreiben & Prüfungsangst

Prüfarzt: PD Dr. T.I. Usichenko, Tel.: 86 5893

Doktorand: Anna Wenzel

Ort der klinischen Studie: Ernst-Moritz-Arndt-Universität Greifswald
Medizinische Fakultät
Klinik für Anästhesiologie und Intensivmedizin
Fleischmannstraße 42-44
D-17487 Greifswald

In dieser Studie untersuchen wir Ihre individuelle Angstreaktion unter Einfluss von Ohrakupunktur und dem Expressiven Schreiben in Prüfungssituationen. Aus der Forschung und klinischen Praxis ist bekannt, dass Ohrakupunktur sowie das Expressive Schreiben angstlindernde Eigenschaften besitzen. Mit unserer Studie möchten wir die Größe des angstlindernden Effekts der Akupunktur am Außenohr wie auch die Größe des angstlindernden Effekts des Expressiven Schreibens untersuchen und die Größen miteinander vergleichen. Die standardisierten Angstempfindungen werden sowohl psychisch, mit dem sogenannten State-Trait-Angstinventar (STAI) und einer visuellen Angstskala (VAS), als auch physisch, durch die Messung der Herzfrequenz, des Blutdruckes sowie der Speichelamylase erhoben. Die Durchführung beider Interventionen erfolgt am Tag vor der Prüfung. Während der Studie wird ein Proband am Expressiven Schreiben, an der Ohrakupunktur oder als Kontrollgruppe an keiner Intervention teilnehmen.

### Was ist Ohrakupunktur?

Die Ohrakupunktur ist eine der Reflextherapien, die über periphere sensorische Stimulation der kranialen Nerven mit Dauernadeln zu den Wirkungen in subkortikalen Hirnarealen führt.

Unerwünschte Wirkungen (Nebenwirkungen) der Akupunktur können sein: Schmerzen beim Einstich oder Entfernen der Nadel, kleine Blutungen, sehr selten (0,001%): Schwindel, Blutdruckabfall, Bradykardie, Tachykardie, psychische Reaktionen, lokal: Chondritis.

**Wie wird die Intervention durchgeführt?**

Die Akupunktur erfolgt am Tag vor der Prüfung mit Dauernadeln, welche bis nach dem Testat im Ohr verbleiben und nach Bedarf eigenständig stimuliert werden können. Je Außenohr werden 5 Nadeln eingesetzt.

**Was ist Expressives Schreiben?**

Das Expressive Schreiben ist ein schriftliches Verfahren, in welchem sich eigenständig sehr emotional und ausdrucksvoll mit den persönlichen Gefühlen und Ängsten hinsichtlich einer bevorstehenden Prüfung auseinandergesetzt werden soll. Mehrere Studien konnten zeigen, dass diese Anwendung signifikant die Prüfungsangst reduzieren, als auch die Prüfungsleistungen verbessern konnten.

**Wie wird die Intervention durchgeführt?**

Dieser Versuch dauert 15 Minuten und findet in einem separaten ruhigen Raum statt. Dabei wird versucht die tiefsten Gedanken und Emotionen bezüglich Ihrer Prüfung in einem Aufsatz zum Ausdruck zu bringen.

**Was ist STAI?**

Das State-Trait-Angstinventar ist eine Methode zur Erhebung von Angst als einen emotionalen Zustand (State) und Angst als Eigenschaft (Trait). Die Zustandsangst ist gekennzeichnet durch Nervosität, Angespanntheit, Besorgtheit, innere Unruhe, Angst vor zukünftigen Ereignissen sowie durch eine erhöhte Aktivität des autonomen Nervensystems. Angst als Eigenschaft bezieht sich demgegenüber auf interindividuelle Differenzen in der Bewertung der Bedrohlichkeit einer Situation.

**Wie wird die Messung durchgeführt?**

Das STAI setzt sich aus einem Fragebogen aus je 20 Fragen zu Zustandsangst und je 20 Fragen zu Angst als Eigenschaft zusammen.

**Was ist VAS?**

Die visuelle Angstskala dient der Erhebung von subjektiven Empfindungen. Es handelt sich bei der VAS um eine horizontale Linie welche durch extreme Endpunkte von 0 (überhaupt nicht ängstlich) bis 100 (vollkommen ängstlich) quantifiziert wird.

**Wie wird die Messung durchgeführt?**

Ziel ist es, seinen subjektiv empfundenen Angstzustand auf dieser Skala von 0 (überhaupt nicht ängstlich) bis 100 (vollkommen ängstlich) durch Markierung mit einem Stift fest zu legen.

**Was ist Speichelamylase?**

Im menschlichen Speichel befindet sich das Enzym α-Amylase. Dieses Enzym ist Produkt der Speicheldrüsen. Die Produktion unterliegt dem sympathischen Nervensystem, wodurch es als sensitiver Biomarker für stressbezogene Ereignisse geeignet ist.

**Wie wird die Messung durchgeführt?**

Es werden drei Proben gewonnen: Am Abend vor der Prüfung, am Morgen der Prüfung, sowie am Abend nach der Prüfung. Der Speichel wird in einem Probengefäß durch einfaches Auffangen gesammelt. Dabei ist darauf zu achten, dass eine Probenentnahme vor dem Verzerr von Nahrung, vor dem Genuss von Getränken und vor dem Zähneputzen zu erfolgen hat.

**Was wird mit den Werten gemacht?**

Die Daten werden im Rahmen einer Doktorarbeit ausgewertet und können Ihnen nicht direkt im Anschluss an den Test mitgeteilt werden. Sind Sie an den ausgewerteten Daten interessiert, können wir Ihnen diese per Email zuschicken.

**Wie lange wird der Test/Versuch dauern?**

Der gesamte Versuch wird drei mal zwei Tage umfassen. Am Tag vor der Prüfung werden wir Ihnen an unserem Standpunkt in der Alten Urologie die Ohrakupunkturnadeln einsetzen oder das Verfahren des Expressiven Schreibens durchführen. Die gesamte Prozedur mit der Erfassung der physiologischen und psychologischen Parameter wird in etwa eine halbe Stunde in Anspruch nehmen. Am Tag der Prüfung bitten wir Sie ein weiteres Mal die

Fragebögen auszufüllen, welches Sie bequem zu Hause erledigen können. Des Weiteren werden wir Ihren Blutdruck und Ihre Herzfrequenz etwa eine halbe Stunde vor Ihrer jeweiligen Prüfung erfassen.

**Untersuchungsablauf**

**Tag 1:** Wir zeigen Ihnen den Untersuchungsraum sowie die Geräte zum Durchführen unserer Untersuchungen. Beginnen werden Sie mit dem Ausfüllen der Fragebögen. Dem schließt sich die Messung der physiologischen Parameter an. Nun erfolgt entweder die Ohrakupunktur, welche durch einen erfahrenen Akupunkteur durchgeführt wird oder Sie werden gebeten sich dem Expressiven Schreiben zu widmen. Abschließend werden noch einmal Herzfrequenz und Blutdruck gemessen. Zu Hause füllen Sie die Fragebögen erneut aus und geben abends eine Speichelprobe ab.

**Tag 2:** Wir bitten Sie am Morgen vor der Prüfung erneut den Speichel aufzufangen und die Fragebögen ein drittes und letztes Mal auszufüllen. Bitte bringen Sie die ausgefüllten Fragebögen und die zwei Probenbehältnisse mit zu Ihrer Prüfung in die Anatomie, wo wir sie einsammeln werden. Des Weiteren werden wir ca. 30 min vor Ihrer Prüfung Ihren Blutdruck und die Herzfrequenz messen. Am Abend nach dem Testat bitten wir Sie ein letztes Mal eine Speichelprobe zu sammeln. Diese kann am Folgetag persönlich in der Anatomie bei einem Studienmitarbeiter abgegeben oder in einer dafür vorgesehenen Sammelbox eingeworfen werden.

## Appendix D

Universitätsmedizin GREIFSWALD

# Expressives Schreiben

**Name:** **Datum:**

**Anleitung:** Wir bitten Sie darum, sich **tiefgründig** mit dem Thema **„Prüfungsangst“** auseinander zu setzen. Lesen Sie sich bitte dazu die Aufgabenstellung gewissenhaft durch. Für den Aufsatz haben Sie genau **15 Minuten** zeit.

Während der heutigen Schreib-Sitzung, sollen Sie sich gehen lassen und über Ihre **tiefempfundenen Gedanken und Gefühle** bezüglich Ihrer Prüfung schreiben. In dem Aufsatz dürfen Sie über Ihre **Gedanken und Gefühle** über die Prüfung selbst, die Auswirkung der Prüfung auf Ihr jetziges Leben und die Folgen der Prüfung für Ihre zukünftigen Ziele schreiben, sowie über alternative Pläne, welche Sie vielleicht haben. Wichtig ist jedoch, dass Sie Ihre **tiefsten Emotionen** hervorholen und sie in Ihrem Aufsatz zum Ausdruck bringen

# Appendix E

**Studienprotokoll (Ohrakupunktur vs. Expressives Schreiben zur Linderung von Prüfungsangst)**

Testat: Name:

| Parameter | vor Intervention<br>TAG 1 | nach Intervention<br>TAG 1 | vor Testat<br>TAG 2 | nach Testat<br>TAG 2 | Bemerkungen |
|---|---|---|---|---|---|
| HF (Schläge/ min) | | | | XXXXXXXXXX | |
| RR | | | | XXXXXXXXXX | |
| STAI X1 | | | | XXXXXXXXXX | |
| STAI X2 | | XXXXXXXXXX | XXXXXXXXXX | XXXXXXXXXX | |
| VAS | | | | XXXXXXXXXX | |
| Speichelamylase<br>• Abgabezeitpunkt<br>• Wert in U/ml | XXXXXXXXXX<br>XXXXXXXXXX<br>XXXXXXXXXX | | | | |
| Testatergebnis<br>• bestanden<br>• nicht bestanden | XXXXXXXXXX<br>XXXXXXXXXX<br>XXXXXXXXXX | XXXXXXXXXX<br>XXXXXXXXXX<br>XXXXXXXXXX | XXXXXXXXXX<br>XXXXXXXXXX<br>XXXXXXXXXX | XXXXXXXXXX<br>XXXXXXXXXX<br>XXXXXXXXXX | |
| Intervention<br>1. Expressives Schreiben<br>2. Ohrakupunktur<br>3. Kontrollgruppe | XXXXXXXXXX<br>XXXXXXXXXX<br>XXXXXXXXXX<br>XXXXXXXXXX | XXXXXXXXXX<br>XXXXXXXXXX<br>XXXXXXXXXX<br>XXXXXXXXXX | XXXXXXXXXX<br>XXXXXXXXXX<br>XXXXXXXXXX<br>XXXXXXXXXX | XXXXXXXXXX<br>XXXXXXXXXX<br>XXXXXXXXXX<br>XXXXXXXXXX | |
| Nebenwirkungen<br>1. Schmerzen beim Einstich<br>2. Schmerzen beim Entfernen der Nadeln<br>3. Schwindel<br>4. Blutdruckabfall<br>5. Bradykardie<br>6. Tachykardie<br>7. psychische Reaktionen<br>8. Chondritis | XXXXXXXXXX<br>XXXXXXXXXX<br>XXXXXXXXXX<br>XXXXXXXXXX<br>XXXXXXXXXX<br>XXXXXXXXXX<br>XXXXXXXXXX<br>XXXXXXXXXX<br>XXXXXXXXXX | XXXXXXXXXX<br>XXXXXXXXXX<br>XXXXXXXXXX<br>XXXXXXXXXX<br>XXXXXXXXXX<br>XXXXXXXXXX<br>XXXXXXXXXX<br>XXXXXXXXXX<br>XXXXXXXXXX | XXXXXXXXXX<br>XXXXXXXXXX<br>XXXXXXXXXX<br>XXXXXXXXXX<br>XXXXXXXXXX<br>XXXXXXXXXX<br>XXXXXXXXXX<br>XXXXXXXXXX<br>XXXXXXXXXX | XXXXXXXXXX<br>XXXXXXXXXX<br>XXXXXXXXXX<br>XXXXXXXXXX<br>XXXXXXXXXX<br>XXXXXXXXXX<br>XXXXXXXXXX<br>XXXXXXXXXX<br>XXXXXXXXXX | |

Hiermit bestätige ich, dass die Daten in das Protokoll korrekt eingetragen sind.

__________ ____________________

Datum Untersucher

Nadeln entfernt am………………………um………………Uhr durch…………………………(Unterschrift)
